# The Nature of Cardiac Remodeling Due to Physical Exercise: More Evidence Towards to the Normal Adaptive Responses of the Heart

**DOI:** 10.5935/abc.20180238

**Published:** 2018-12

**Authors:** Lucas Helal, Anderson Donelli da Silveira

**Affiliations:** 1 Laboratório de Fisiopatologia do Exercício (LaFiEx) - Hospital de Clínicas de Porto Alegre - Programa de Pós-Graduação em Cardiologia e Ciências Cardiovasculares - Faculdade de Medicina - Universidade Federal do Rio Grande do Sul, Porto Alegre, RS - Brazil; 2 Grupo de Cardiologia do Exercício (CARDIOEX) - Hospital de Clínicas de Porto Alegre - Programa de Pós-Graduação em Cardiologia e Ciências Cardiovasculares - Faculdade de Medicina - Universidade Federal do Rio Grande do Sul, Porto Alegre, RS - Brazil

**Keywords:** Ventricular Remodeling, Atrial Remodeling, Cardiac Remodeling, Exercise, Exercise Movement Techniques, Resistance Training, Running, Weight Lifting, Blood Pressure, Arrhythmias

Cardiac remodeling due to exercise training overload (the so-called "athlete's heart") has been widely investigated since the 70's and is still acknowledged by the scientific community. To differentiate normal adaptive responses ("benign") from abnormal ones remains a challenge. Here we address some important issues related to the discussion on cardiac remodeling in exercise, recently revisited by Vidaletti-Silva and colleagues^1^ in this issue.

In terms of morphological adaptations, the left atrial and ventricular hypertrophy call attention due to their potential association with the onset of supraventricular tachyarrhythmias^2^ and also of ventricular arrhythmias,^3^ which may result in undesirable events.^4^

However, mounting evidence has been suggesting that the remodeling response due to exercise training load (e.g., length of exposure, intensity, modality etc.) may not configure a state of disease - i.e., the "physiological but nor pathological" remodeling.^5^"

With a focus on the left chambers, it is known that the blood pressure and the volumetric overload may result in two classical morphological characteristics, accordingly to the Morganroth hypothesis^6^ - an increase of the left cavities' volume for those overloaded by the cardiac output (i.e., endurance athletes); or the hypertrophy of the left ventricle (LV) septum for those overloaded by blood pressure levels (i.e., strength-trained athletes). As a minor comment, the Morganroth hypothesis has been recently revisited after the observation of cases of septal hypertrophy in endurance athletes.^7^

Although well-established in scientific literature, Vidaletti-Silva et al.^1^ have addressed the question of differences in cardiac remodeling due to sports modalities through a cross-sectional, comparator-group design, comparing endurance athletes (i.e., runners) and strength-trained athletes (i.e., powerlifters) - two classes and levels of modalities that seem appropriate for this comparison. In their findings, no moment-differences between groups were observed for the LV mass, when adjusted for their surface area. As expected, septal and posterior LV thickness were different between endurance and strength athletes, but not the LV end-diastolic volume. The vascular function (i.e., flow-mediated dilation and peripheral vascular resistance) was also evaluated and no differences were found. The take-home message of this study, at least in light of our interpretation, is that athletes in a range of 5 to 7 years of training have adaptations no bigger than the established thresholds for normality for LV dimensions^8^ and wall thickness.^9^

We should acknowledge that, even within borderline values, there were no impairment of the systolic and diastolic function of the myocardium in either groups, depicting the normal adaptive nature of the cardiac structure findings. Even though a simple experiment, this cross-sectional study corroborates the hypothesis of different cardiac adaptations due to different training modalities. Finally, to detect some abnormal morphological adaptations in athletes remains a challenge, especially for those within borderline values. We welcome studies such as this one - that sheds a light on this gray area.

## References

[1] Silva DV, Waclawovsky G, Kraemer AB, Stein C, Eibel B, Grezzana GB (2018). Comparison of cardiac and vascular parameters in powerlifters and
long-distance runners comparative cross-sectional study. Arq Bras Cardiol.

[2] Pelliccia A, Maron BJ, Di Paolo FM, Biffi A, Quattrini FM, Pisicchio C (2005). Prevalence and clinical significance of left atrial remodeling in
competitive athletes. J Am Coll Cardiol.

[3] Ghali JK, Kadakia S, Cooper RS, Liao YL (1991). Impact of left ventricular hypertrophy on ventricular arrhythmias
in the absence of coronary artery disease. J Am Coll Cardiol.

[4] John RM, Tedrow UB, Koplan BA, Albert CM, Epstein LM, Sweeney MO et al (2012). Ventricular arrhythmias and sudden cardiac death. Lancet.

[5] Maron BJ (2009). Distinguishing hypertrophic cardiomyopathy from athlete's heart
physiological remodelling clinical significance, diagnostic strategies and
implications for preparticipation screening. Br J Sports Med.

[6] Morganroth J, Maron BJ, Henry WL, Epstein SE (1975). Comparative left ventricular dimensions in trained
athletes. Ann Intern Med.

[7] Lewis EJH, McKillop A, Banks L (2012). The Morganroth hypothesis revisited endurance exercise elicits
eccentric hypertrophy of the heart. J Physiol.

[8] Pellicia A, Di Paolo FM, Maron BJ (2002). The athlete's heart remodelling, electrocardiogram and
preparticipation screening. Cardiol Rev.

[9] Pelliccia A, Maron BJ, Spataro A, Proschan MA, Spirito P (1991). The upper limit of physiologic cardiac hypertrophy in highly
trained elite athletes. N Engl J Med.

